# Crystal structure of a new benzoic acid inhibitor of influenza neuraminidase bound with a new tilt induced by overpacking subsite C6

**DOI:** 10.1186/1472-6807-12-7

**Published:** 2012-05-06

**Authors:** Lalitha Venkatramani, Eric S Johnson, Gundurao Kolavi, Gillian M Air, Wayne J Brouillette, Blaine HM Mooers

**Affiliations:** 1 Department of Biochemistry and Molecular Biology, University of Oklahoma Health Sciences Center, 941 Stanton L. Young Blvd, Oklahoma City, OK, 73104, USA; 2 Department of Chemistry and Center for Biophysical Sciences and Engineering, University of Alabama at Birmingham, 901 14th Street South, Birmingham, AL, 35294, USA

**Keywords:** Influenza neuraminidase inhibitor, Enzyme-ligand complex, Antiviral, Structure-based drug design, Glycoprotein, Glycan structure, Influenza virus, Benzoic acid, Pyrrolidinone

## Abstract

**Background:**

Influenza neuraminidase (NA) is an important target for antiviral inhibitors since its active site is highly conserved such that inhibitors can be cross-reactive against multiple types and subtypes of influenza. Here, we discuss the crystal structure of neuraminidase subtype N9 complexed with a new benzoic acid based inhibitor (**2**) that was designed to add contacts by overpacking one side of the active site pocket. Inhibitor **2** uses benzoic acid to mimic the pyranose ring, a bis-(hydroxymethyl)-substituted 2-pyrrolidinone ring in place of the *N*-acetyl group of the sialic acid, and a branched aliphatic structure to fill the sialic acid C6 subsite.

**Results:**

Inhibitor **2** {4-[2,2-bis(hydroxymethyl)-5-oxo-pyrrolidin-1-yl]-3-[(dipropylamino)methyl)]benzoic acid} was soaked into crystals of neuraminidase of A/tern/Australia/G70c/75 (N9), and the structure refined with 1.55 Å X-ray data. The benzene ring of the inhibitor tilted 8.9° compared to the previous compound (**1)**, and the number of contacts, including hydrogen bonds, increased. However, the IC_50_ for compound **2** remained in the low micromolar range, likely because one propyl group was disordered. In this high-resolution structure of NA isolated from virus grown in chicken eggs, we found electron density for additional sugar units on the N-linked glycans compared to previous neuraminidase structures. In particular, seven mannoses and two N-acetylglucosamines are visible in the glycan attached to Asn200. This long, branched high-mannose glycan makes significant contacts with the neighboring subunit.

**Conclusions:**

We designed inhibitor **2** with an extended substituent at C4-corresponding to C6 of sialic acid-to increase the contact surface in the C6-subsite and to force the benzene ring to tilt to maximize these interactions while retaining the interactions of the carboxylate and the pyrolidinone substituents. The crystal structure at 1.55 Å showed that we partially succeeded in that the ring in **2** is tilted relative to **1** and the number of contacts increased, but one hydrophobic branch makes no contacts, perhaps explaining why the IC_50_ did not decrease. Future design efforts will include branches of unequal length so that both branches may be accommodated in the C6-subsite without conformational disorder. The high-mannose glycan attached to Asn200 makes several inter-subunit contacts and appears to stabilize the tetramer.

## Background

Influenza A viruses display two membrane-anchored glycoproteins, hemagglutinin (HA) and neuraminidase (NA). HA mediates attachment of the virus to sialic acid receptors on host cells to initiate virus infection. After virus replication, NA removes sialic acid residues from viral and cellular glycoproteins to facilitate virus release and allow spread of infection to new cells. In the absence of NA activity, the progeny virions aggregate and infection ends [1,2].

The distinct antigenic properties of HA and NA from different viruses are used to classify influenza type A into subtypes—16 for HA and 9 for NA. H1N1 (1918), H2N2 (1957) and H3N2 (1968) caused the major influenza pandemics of the 20^th^ century. Evidence that the new HA and sometimes NA genes originated in wild birds before appearing in humans has raised concerns about the recent spread of highly pathogenic avian H5N1 viruses. These viruses have the potential to gain transmissibility among humans and thereby devastate immunologically naïve human populations [3]. Type B viruses are not carried by birds and are not divided into antigenic subtypes, but since the mid-1980s, two lineages, B/Victoria and B/Yamagata, have been evolving concurrently in humans [4].

**Figure 1:**
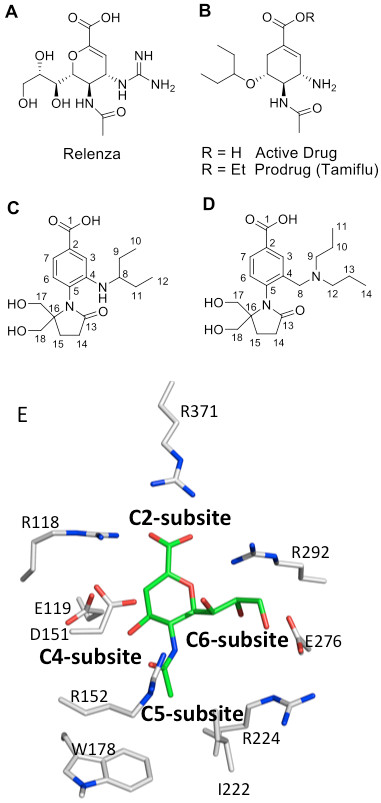
**NA inhibitors.** A, Zanamivir; B, oseltamivir; C, Inhibitor 1 (compound 14 of Brouillette et al. [17]); D, Inhibitor **2**; E, The NA subsites that surround the transition state analog 2-deoxy-2-dehydro-N-acetylneuraminic acid (DANA). Note that in the benzoic acid series, subsite 6 is adjacent to C4 of the benzene ring. Figure made with PDB ID: 1NNB.

NA is a good target for structure-based enzyme inhibitor design because its well-characterized active site is conserved across all influenza A and B viruses [5]. The 11 amino acids in the active site that interact with sialic acid remain unchanged amidst extensive genetic variation in the rest of the sequence [6-8]; for an alignment, see [9]. Structure-based drug design led to the successful antiviral drugs zanamivir (GG167) [10] and oseltamivir (GS4071) [11] that are effective against different types and subtypes of influenza. Zanamivir is based on a transition state analogue of sialic acid while oseltamivir replaces sialic acid's pyranose ring with a cyclohexene scaffold (Figure 1). The high potency and oral activity of oseltamivir encourages the use of alternative scaffolds to replace the sugar ring of sialic acid [12-14]. Benzene ring scaffolds minimize the number of chiral centers, potentially simplifying the chemical synthesis and reducing associated expenses [15-18]. The challenge is to configure substituents on the ring to optimize the interactions with the active site. The direct contacts with sialic acid are conserved in all influenza NAs, but the subsites that surround the sialic acid—named according to the ring atoms of sialic acid (Figure 1E)—show differences in size and shape between strains and sometimes account for large differences in potency between N1, N2 and B NAs [17,19]. In addition, inhibitors with a hydrophobic side chain often show differences in potency between the two structural groups of type A NA: group 1 (subtypes N1, N4, N5 and N8) and group 2 (subtypes N2, N3, N6, N7, and N9) [20].

X-ray structures of several simple benzoic acid derivatives bound to influenza B NA show that these inhibitors bind to the active site in the same orientation as sialic acid [15,16]. The derivatives' common carboxylate substitution at the C2 position maintains the native interaction with the arginine triad (Arg292, Arg118, and Arg371 in N2 numbering), and the N-acetyl substitution at the C5 subsite maintains the native interaction with Arg152.

In addition, using a benzene ring scaffold, hydrophobic groups of varying lengths fit into the glycerol binding site (C6 subsite) of the sialic acid [21]. The length and branching of the aliphatic chain have been modified to improve inhibition, but benzoic acids from this structural class have yet to match the inhibitory activities observed for zanamivir and oseltamivir. It is likely that this results from the lack of a basic group (aliphatic amine or guanidine) in the C4 subsite, which forms an important salt bridge with inhibitors in clinical use.

One of the best compounds from the existing benzoic acid series, inhibitor **1**, (Figure 1C) lacks the C4 subsite substituent yet exhibited moderately potent (low to mid nM) activity against NAs of the N2 and N9 subtypes of influenza A virus, with less inhibitory activity against influenza B virus NA, as observed for other compounds with a similar hydrophobic substituent on the benzene ring [18].

We are trying to broaden the specificity of the benzoic acids against influenza viruses of different subtypes and to increase potency. The best way to accomplish this goal is likely by adding a basic substituent to form a salt bridge in the C4 subsite. However, attempts to design substituents on the benzene ring that occupy both the negatively charged C4 subsite and the glycerol binding site (C6 subsite) of sialic acid have been difficult because the two subsites are offset from the plane of the benzene ring. The results of molecular modeling studies using *FlexX*[22] suggested that a different “tilt” of the benzene ring may be needed, and one way to alter the tilt may be to change the size of the hydrophobic substituent, such that an increase or a decrease in steric crowding reorients the benzene ring in the binding site.

The inhibitor studied here, compound **2**, like compound **1**, contains no chiral centers but has a longer hydrophobic substituent consisting of a 3-heptyl (instead of 3-pentyl) group positioned one atom further from the benzene ring (Figure 1D). Enzyme inhibition assays indicate that the affinity of **2** is in the low micromolar range for type A NAs but, compared to **1**, lower for type B NA (Table 1) [12,17]. We have determined the crystal structure of **2** in complex with a type A N9 NA to a resolution of 1.55 Å, and we discuss it in terms of the successful reorientation of the benzene ring and the lack of improvement in binding affinity. The structure of the complex suggests routes to design inhibitors that might show improved affinity.

**Table 1:** **IC**_**50**_**values of the inhibitor 2 compared with compound 1**

**Inhibitor**	**NA**	**IC50 (μM)**
**1**[17]	A/Udorn/72 NA (N2)	0.49
	A/tern/Australia/G70c NA (N9)	4.4
	B/Lee/40 NA	271
**2**[23]	H3N2	2
	H1N1	20
	A/tern/Australia/G70c NA (N9)	9.1 ± 2.2
	B/Lee/40 NA	180

## Results and discussion

### Overall structure

N9 NA crystals that had been soaked with inhibitor **2** showed similar unit cell dimensions and space group symmetry as low temperature crystal structures of other N9-inhibitor complexes and the uncomplexed mutant R292K (Protein Data Bank identifier [PDB ID] 2QWA) [24]. The available uncomplexed native structures (PDB ID, 1NNA and 7NN9) were determined at room temperature and were not as suitable for direct comparison with our low temperature data, so we judged the crystal structure of the uncomplexed mutant R292K to be a less biased starting model for molecular replacement. Inhibitor **2** bound to the active site with nearly full occupancy (refined occupancy factor of 0.68) and did not bind to additional sites such as the second sialic acid binding site [25,26] (PDB ID, 2C4A).

### Inhibitor–N9 interactions

**Figure 2:**
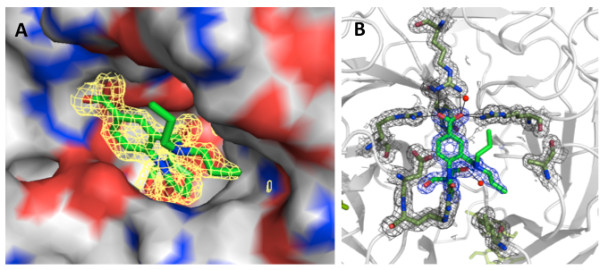
**Electron density of inhibitor 2 in the active site. A, 2*F***_**o**_**-*F***_**c**_**map of inhibitor contoured at 1σ shows inhibitor fitting snugly in the active site cavity; B, 2*F***_**o**_**-*F***_**c**_**map of inhibitor and interacting amino acids of the NA.**

The X-ray data were of high quality and the structure of **2** complexed with N9 NA was refined with 1.55 Å X-ray data. The final model had good geometry (Table 2) with all amino acid residues in the allowed region of the Ramachandran plot (96.1*%* in the favored region and 0% outliers). Initial F_o_–F_c_ maps showed clear electron density of the inhibitor in the active site of NA (Figure 2A). The two substituents of the inhibitor's pyrrolidine ring were buried inside the active site cavity (dihedral angle C6-C5-N5-C13 at the atropisomeric center −112°) with no indication of alternative conformers with rotation about the C8-N bond. The F_o_–F_c_ maps revealed very good electron density for the propyl group involving C9, C10 and C11, but the other propyl group involving C12, C13 and C14 was disordered (Figure 2), showing two tracks of weak electron density. We tried to refine this branch with split occupancy, but the electron density after refinement was not continuous. This result suggested that this branch adopted additional conformations and that each of the two tracks of weak electron density had much less than 50% occupancy. At this point, we decided to model this branch in one partially occupied conformation rather than all of the possible conformations.

**Table 2:** X-ray diffraction data and refinement statistics

**A. X-ray Data**	
X-ray Source	SSRL
	Beam line 7–1
Space Group	*I*432
Cell Dimensions	
a = b = c (Å)	181.0
Asymmetric Unit	1 monomer
Resolution Range (Å)	27.9–1.55
	(1.63–1.55)
*R*_merge_	0.067
	(0.767)*
*R*_p.i.m._*	0.013
	(0.25)
mean (I/σ(I))	29.4
	(3.8)
Multiplicity	24.7
	(17.8)
No. Unique Reflections	72,486
	(10,350)
Data Completeness (%)	99.9
	(99.1)
B. Refinement	
*R*_*work*_	0.1267
*R*_*free*_	0.1552
Bonds (Å)	0.008
Angles (˚)	1.562
No. Amino Acids	391
No. Glycan monomers	15
No. D-glucose molecules	4
No. Waters	458

(Statistics for the highest resolution shell are in parentheses).

*R_*merge*_ is high in the highest resolution shell due to the high multiplicity of the data. The *R*_*p.i.m.*_ is independent of the data multiplicity and shows that the data in the highest shell have a reasonable discrepancy of 25%. *R*_*p.i.m.*_is the precision indicating *R*_*merge*_[27].

Potential interactions between inhibitor **2** and N9 NA were assessed using *Chimera*[28]. In addition, potential hydrogen bonds as well as hydrophobic contacts were identified with *HBPLUS*[29] (see Table 3 for the geometric criteria). Favorable hydrophobic contacts were defined as non-bonded contacts between two carbon atoms at a distance of ≤4 Å [30].

**Table 3:** Contacts observed between inhibitor 2 and N9 NA; 3LV is the residue name assigned to the inhibitor by the PDB and 488 is the residue number of the inhibitor

**Inhibitor atom**	**N9 atom**	**distance (Å)**	**Angle(D-A-AA)°**	
Potential hydrogen bonds^1^				
3LV488-O1	Arg118-NH1	2.84	117.3	
3LV488-O1	Arg371-NH1	2.86	112.8	
3LV488-O2	Arg292-NH1	3.34	97.1	
3LV488-O2	Arg292-NH2	3.25	101.2	
3LV488-O2	Arg371-NH2	2.84	124.9	
3LV488-O2	HOH-747	2.85		
	HOH-747	3.25		Asn347 OD1
3LV488-O15	Arg152- NH1	2.54	145.9	
3LV488-O20	HOH-612	2.72		
	HOH-612	2.82, 3.16		Glu227 OE2, Thr225 O
Van der Waals contacts^2^ (≤4.0 Å)				
3LV488-C1	Tyr406-OH	2.97	119.4	
	Arg371-NH1	3.70	96.2	
	Arg371-NH2	3.49	106.5	
	Tyr406-CZ	3.83	42.5	
3LV488-O1	Tyr406-OH	3.50	99.8	
	Arg118-CZ	3.65	43.7	
	Arg118-NH2	3.56	83.0	
	Arg371-CZ	3.59	47.2	
	Arg371-NH2	3.45	85.5	
3LV488-C2	Tyr406-OH	2.80	136.4	
3LV488-O2	Tyr406-OH	3.32	123.9^3^	
	Arg371-CZ	3.76	38.3	
	Arg371-NH1	3.82	96.2	
3LV488-C3	Tyr406-OH	3.19	161.8	
3LV488-C6	Glu119-OE2	3.39	119.6	
	Asp151-CG	3.49	74.6	
	Asp151-OD1	3.37	84.4	
	Asp151-OD2	3.87	62.7	
3LV488-C7	Glu119-OE2	3.49	136.6	
	Asp151-OD1	3.52	97.7	
	Asp151-CG	3.89	63.8	
	Tyr406-OH	3.23	118.0	
	Arg118-NH1	3.63	113.4	
3LV488-C10	Ile222-CD1	3.63	122.8	
3LV488-C11	Ala246-CB	3.90	115.5	
	Ile222-CD1	3.54	105.5	
	Ile222-CB	3.88	87.6	
	Arg224-CZ	3.43	79.4	
	Arg224-NE	3.78	64.8	
	Arg224-NH1	3.44	78.4	
	Arg224-NH2	3.81	63.4	
	HOH-945	2.82	---	
3LV488-C14	Arg292-NH2^4^	3.50	140.0	
3LV488-O15	Asp151-CB	3.59	100.5	
	Arg152-CG	3.84	73.6	
	Arg152-CD	3.71	83.0	
	Arg152-CZ	3.70	22.6	
3LV488-C16	Trp178-CE3	3.59	91.2	
	Trp178-CZ3	3.88	67.8	
	Trp178-O	3.78	135.7	
	Arg152-CD	3.73	78.1	
	Arg152-CG	3.73	78.1	
3LV488-C17	Trp178-O	3.43	139.6	
	Glu227-OE2	3.62	135.8	
3LV488-C19	Trp178-O	3.19	167.7	
	Glu119-CD	3.88	85.8	
3LV488-O19	Asp151-O	3.26	116.7	
	Asp151-CB	3.73	97.2	
	Arg156-NH1	3.68	139.0	
	Trp178-O	2.82	162.5	
3LV488-C20	Glu227-CG	3.88	74.3	
	Glu227-CD	3.77	55.7	
	Glu227-OE2	3.23	105.4	
	Glu277-OE2	3.34	140.2	
3LV488-O20	Glu227-OE2	3.68	115.2	
	Glu277-CG	3.80	70.4	
	Glu277-CD	3.59	31.9	
	Glu277-OE2	2.61	133.4	
Water bridge – inhibitor – Glu276				
3LV488-O20	HOH-612	2.72		
612-HOH	553-HOH	2.64		
553-HOH	Glu276-OE1	2.82	142.2	
Glu276 interactions				
Glu276-OE1	Arg224-NE	2.72	114.2	
Glu276-OE2	Arg224-NE	3.50	83.2	
Glu276-OE2	Arg224-NH2	2.81	115.9	
Glu276-OE2	His274-NE2	2.73	119.7	

^1^Potential hydrogen bonds were identified using *HBPLUS*[29]. Potential hydrogen bonds were interactions between donor and acceptor atoms that met the following geometric requirements: (i) a donor acceptor distance (D-A) < 3.5 Å and a hydrogen acceptor distance (H-A) < 2.5 Å and (ii) a donor-acceptor-acceptor antecedent angle (D-A-AA), of > 90° [31]**.**

^2^Favorable hydrophobic contacts were defined as non-bonded contacts between two carbon atoms at a distance of ≤4 Å [28].

^3^Freely rotating hydroxyl hydrogen atoms had the potential to form hydrogen bonds with two alternative acceptor atoms. Only the more probable acceptor atom at the shorter distance from the hydrogen atom was reported by *HBPLUS.*

^4^There were several conformations of the propyl chain C12-C14. We used the best, but it is of low occupancy so the interactions involving C14 contribute very little.

**Figure 3:**
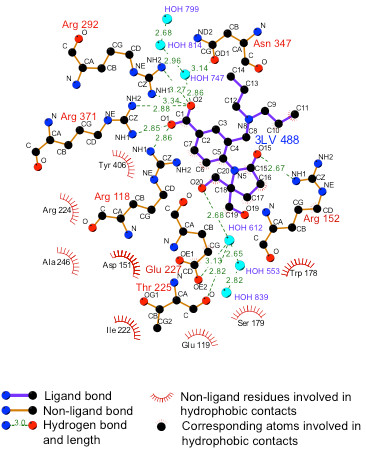
**Interactions between compound 2 and NA.*Ligplot +*** [34]**diagram showing interactions.**

Structural studies of complexes between several benzoic acid leads and type B NA showed that these inhibitors were bound in the active site in a similar fashion to sialic acid [21,32], and the same is observed in the new structure (Figure 3). The carboxylate group of the inhibitor interacts with the guanidinium groups of arginine residues at positions 118, 292, and 371 as seen in all NA substrate and inhibitor complexes and calculated to be energetically important [33]. A weak hydrogen bond is seen between Tyr406 and the carbonyl oxygen that interacts with Arg292. The pyrrolidine ring interacts with Arg152 by forming a hydrogen bond with its carbonyl oxygen (O15). The methylene group C16 lies in the hydrophobic pocket formed by Trp178 and Arg152. The methylene group C17 is involved in C-H^…^O bonds with Trp178 carbonyl O and Glu227 OE2. One of the hydroxyl methyl groups (O20) is hydrogen bonded to Glu277. Atom O20 also interacts with Glu276 through two water molecules, HOH553 and HOH612, by a chain of hydrogen bonds. The other hydroxyl methyl group (O19) was directed toward Trp178 O and Glu119 OE2. The one well-ordered propyl chain is anchored by hydrophobic contacts with Ile222, Ala246, and Arg224. The other propyl chain that is disordered is exposed to the solvent and therefore not contributing to binding energy because the one interaction seen (Table 3) is of low occupancy and at the upper limit of the distances considered as significant.

### Comparative analysis

The model of **2** complexed with N9 NA was compared to sialic acid complexes with A/Tokyo/3/1967 N2 NA, PDB ID: 2BAT [35] and A/tern/Australia/G70c/75 N9 NA, PDB ID: 1MWE [26]. When our new structure was fitted with CCP4's *SUPERPOSE*[36] using the 11 active site residues, the root mean square deviations (RMSDs) were 0.7 Å to N2 and 0.4 Å to N9. The model gave a RMSD of 1.65 Å when superposed on the complex of compound **1** with B/Lee/40 NA [21] using *COOT* secondary structure matching [37]. These small RMSDs suggest that replacing the sialic acid ligand with the inhibitor did not disturb the orientation of the active site residues of NA. The larger deviation between N9 and B NAs was expected given that there is less than 30% sequence identity. In all the above comparisons, most of the active site residues (Asn151, Arg152, Glu227, Arg371, Arg292 and Arg118—numbering as in the current complex) superposed well in the two molecules and a maximum shift of 0.2 to 0.5 Å was observed. However, the side chain of Glu276 showed significant conformational change in the current complex when compared to NA-sialic acid or NA-zanamivir complexes. The two oxygen atoms OE1 and OE2 of Glu227 in the current complex moved toward the solvent and away from the active site by 1 Å. In this position, the carboxyl group interacted with NE of Arg224 and NH2 of His274. Hence, Glu276 did not form the direct hydrogen bonds with the inhibitor hydroxyl oxygen O20 analogous to those that Glu276 formed with the glycerol side chain of sialic acid and its transition state mimics. However, O20 of the inhibitor was linked to Glu276 through the water molecules HOH552 and HOH611. The C14 atom of the inhibitor is seen to make a hydrophobic contact with Glu276 but the low occupancy of the C12-C14 chain precludes a significant contribution to binding.

In the compound **1** complex with influenza B NA [21], the aliphatic chain forms van der Waals contacts with the side chains of Arg292, Asn294 and Glu275 while the hydroxymethyl groups interact with Glu117, Trp177 and Glu276. The rotation of the Glu276 side chain towards Arg224 observed in our complex was noted in the other structures where the inhibitor carries a hydrophobic side chain [21]. N1 NAs have additional flexibility compared to N9 in the 150 loop but binding of oseltamivir to wild-type N1 NA involves a conformational change in the side chain of Glu276 relative to the ligand free enzyme [20,38] similar to that seen in N9 NAs.

**Figure 4:**
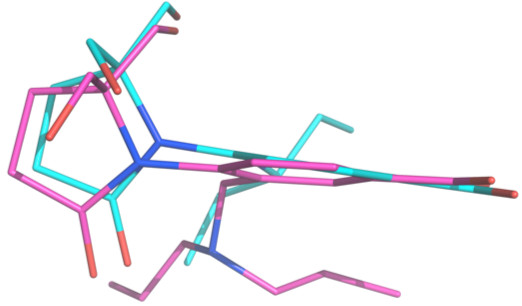
**Bound configurations of Compound 1 (PDB ID 1B9V; cyan) compared to compound 2 (magenta).** The proteins of the complex structures were aligned using *DaliLite*[40]. The view is in the plane of the benzene ring of **1**, showing that the benzene ring of **2** is slightly rotated as well as tilted 8.9° compared to **1**.

We compared the NA and inhibitor contacts with previously reported benzoic acid inhibitor-NA structures using *Chimera* with the relatively stringent constraint of distance ≤3.5 Å and including both polar and hydrophobic contacts. In the BANA 113-B NA complex [15][39], 12 drug atom made 21 contacts ≤3.5 Å with 10 amino acids of NA. In **1**-B NA [21], 14 drug atoms make 23 contacts with 13 amino acids. Inhibitor **2** shows a small increase to 15 drug atoms making 28 contacts with 12 amino acids. The benzene ring of **2** is tilted by 8.9° relative to compound **1** (Figure 4), increasing the number of contacts as was predicted in the design. However, one branch of the 3-heptyl group makes no significant contacts due to multiple conformations, which may be why the IC50 is no better than the previous compounds (Table 1).

Inhibitor **2** did not bind to the second sialic acid binding site observed in the structure of N9 complexed with sialic acid at low temperature [26] (PDB ID, 1MWE) or at room temperature (PDB ID, 2CML).

### Glycan structures

**Figure 5:**
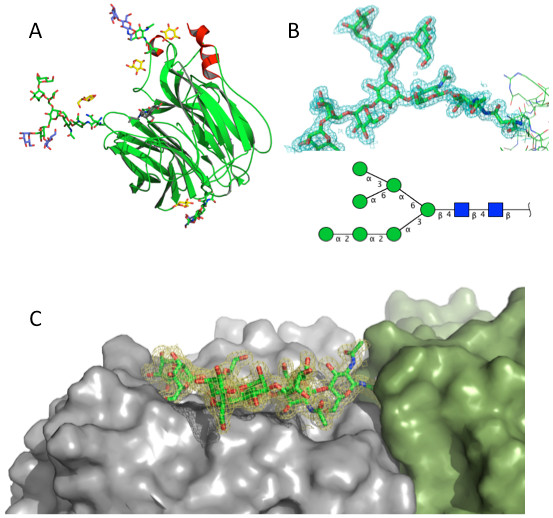
**A. Carbohydrates in the N9-compound 2 model at 1.55 Å resolution.** Carbon atoms are green for N9 NA and the N-linked glycans attached at positions 86, 146 and 200 that are in common with other structures. Mannose residues not previously seen in N9 structures (blue) and the four glucose molecules from the cryoprotectant (yellow) are shown. Compound **2** is colored gray. **B.** Electron density (2*F*_o_*F*_c_ at 1σ) for the glycan at Asn200. Also shown is the cartoon of the glycan structure using standard Consortium for Functional Glycomics symbols (green circle, mannose; blue square, *N*-acetylglucosamine) made with Glycan Builder [45]. **C.** Interaction of the glycan attached to Asn200 (green) with the neighboring subunit (gray) built by applying crystallographic symmetry.

When compared to the structure of the N9 mutant R292K (PDB ID, 2QWA) and the 1.4 Å resolution structures of native N9 in complex with other inhibitors (PDB IDs, 1F8D and1F8E), our structure has four additional sugar units in the glycan chains attached to the delta nitrogen atoms of Asn146 and Asn200 (N2 numbering) (Figure 5). At site 146, we found a second NAG residue and evidence for a β-D-mannose (BMA). At site 200, we found two additional mannose residues. One mannose (Man477) is bonded to the O6 oxygen of Man475G. The second mannose (Man476H) is linked to the O3 oxygen atom of Man475G, resulting in a GlcNAc_2_-Man_7_ structure (Figure 5B). This glycan attached to Asn200 contacts 11 amino acids of the neighboring subunit when the tetramer is built by symmetry (Table 4 and Figure 5C). A high-mannose glycan at Asn200 is also present in N2 (A/Tokyo/67) NA, where it interacts with the adjacent symmetry-related subunit at amino acids 391–394 and 453–455 (PDB ID, 1NN2). This glycan forms part of the Mem5 monoclonal antibody epitope on the 1998 N2 NA, and its interactions with the adjacent subunit are not disturbed by the presence of the antibody [41], suggesting the subunit interactions are energetically significant. In N9 there is experimental evidence that the glycan attached to Asn200 contributes to folding or oligomerization because the mutation N200L in N9 NA resulted in 80% reduction in enzyme activity although the expression level was the same as wild type [42]. In both N2 and N9 this subunit-spanning glycan is of the high mannose type, as is the glycan at 86 in N2 [43]. N6 NA (PDB ID 1V0Z) crystallized with the tetramer as the asymmetric unit, so there is direct evidence for the inter-subunit interaction of the glycan attached to Asn200 in N6 NA. N1 NAs do not have a predicted glycosylation site at 200, but a study of N1 NA assembly suggested that tetramerization requires high-mannose glycans. When all the glycans were processed to complex structures then the resulting dimers and monomers did not assemble into tetramers [44]. The PDB structures of N1 NA have no density for sugar residues, so it is not known if one of the other glycans is a high-mannose structure that spans the subunit interface in the same way as the glycan at Asn200 in N9, N2 and N6.

**Table 4:** Contacts <3.5 Å between the high mannose glycan at Asn200 and the adjacent subunit built as a symmetry-related monomer

**Sugar**	**Sugar atom**	**Amino acid**	**Atom**	**Distance (Å)**
NAG469A	O5	G454	CA	3.39
		Q455	N	3.35
	C6	L453	O	3.43
	O6	Q455	CG	3.25
NAG 470B	O3	G394	N	2.96
	C3	G394	O	3.16
BMA 471 C	O2	G394	CA	3.21
MAN 472D	O3	R364	NH2	3.02
	C3	E375	OE1	3.39
	O3	E375	OE2	2.58
	O4	E375	OE1	2.75
MAN 473E	O5	R364	NH2	3.15
	O6	K389	NZ	2.82
	O6	D330	OD2	2.54
MAN 473 F	O2	N329	OD1	3.10
	O3	D330	N	2.93
	O3	N329	OD1	2.74
	O3	N329	CA	2.99
	O4	R327	NH2	3.11
	O6	I366	O	2.75

### Glucose molecules

We used 49% (w/v) glucose as cryoprotectant, and we observed four glucose molecules bound with greater than 50% occupancy in the structure of the inhibitor complex (Figure 4A). The glucose molecules are in a mix of alpha and beta configurations about the anomeric carbon atom. The O3 oxygen atom of Glc487 interacts with the ND2 nitrogen atom of Arg141 through a hydrogen bond. O3 and O1 of Glc486 forms hydrogen bonds with NE2 of Gln315 and ND2 of Asn338 respectively.

## Conclusions

Our aim in designing inhibitor **2** was to extend the substituent at C4, corresponding to C6 of sialic acid, to (i) increase the contact surface in the C6-subsite and (ii) force the benzene ring to tilt to maximize these interactions while retaining the interactions of the carboxylate and the pyrolidinone substituents. The crystal structure at 1.55 Å shows that we were partially successful in that the ring in **2** is tilted relative to compound **1** (Figure 4) and the overall number of contacts is increased. The IC_50_ did not decrease, and the reason became clear when we solved the crystal structure. The second propyl group is not making any contacts but is freely waving above the surface of the NA. Future design efforts will include unequal branches that may be accommodated in the C6-subsite.

The N9 NA that was crystallized for this experiment was purified from virus grown in embryonated chicken eggs, so it contains a full complement of vertebrate processed N-linked glycans, in contrast to NA expressed in insect cells which has truncated glycan structures. We therefore refined the glycans as far as we could see electron density. Most glycans are flexible and only the first few sugars are seen, but the glycan attached to Asn200 is well resolved due to interaction with the adjacent subunit and is seen to contain GlcNAc_2_Man_7_. Mutation data suggests this glycan plays a role in stabilizing the NA tetramer, which is a significant property because, for reasons that are not understood [9], only the NA tetramer has enzymatic activity.

## Materials and methods

### Inhibitor synthesis

The synthesis, purification and evaluation of the in vitro inhibitory activity of **2** {4-[2,2-bis(hydroxymethyl)-5-oxo-pyrrolidin-1-yl]-3-[(dipropylamino)methyl]benzoic acid} will be described elsewhere [23].

### Protein preparation and crystallization

The reassortant virus A**/**NWS/33_H_-A/tern/Australia/G70C/75_N_ (H1N9) was grown and purified as previously described [25]. The purified virus was digested with Pronase (Sigma) at a concentration of 6 mg/ml at 37 C for 16 hours. The cores were removed by centrifugation, and the released heads were pooled, concentrated, and purified by gel filtration using FPLC [41]. The purified protein was concentrated to 10 mg/ml. Single crystals of N9 NA were grown by vapor diffusion using the hanging drop method containing equal volumes of N9 and the reservoir solution of 1.9 M potassium phosphate buffer pH 6.9 [8].

### Inhibitor soaking, cryoprotection and X-ray data collection

The successful flash cooling of large (0.2 − 0.4 mm per edge) cubic crystals in liquid nitrogen required serial equilibration by vapor diffusion. The estimated osmolality for 1.9 M K-phosphate buffer (pH 6.9) in the reservoir solution was matched to that of glucose using standard tables [46,47]. Crystals were placed over wells with 200 mM K-phosphate buffer (pH 6.9) containing 45 g/100 ml (45% w/v) glucose for 6 hours to two days and then placed over a well with 46% glucose. This procedure was repeated until the crystals were over wells that contained 49% glucose. After the final equilibration, the crystals were soaked in 49% glucose and 200 mM K-phosphate with 25 mM inhibitor for 5 minutes before they were mounted in a rayon cryoloop and vitrified at 100 K in a nitrogen cold-stream.

X-ray data were collected at 100 K at SSRL beam line 7–1 on an ADSC Quantum 315 CCD detector using monochromatic radiation with a wavelength of 0.9537 Å. The X-ray data were collected at distances of 300 and 220 mm with oscillation angles of 0.5° and 0.35° respectively and with 2 s exposures at both distances.

The X-ray data were indexed and integrated with *XDS*[48]. The integrated intensities were scaled and merged with the *CCP4*[49] program *SCALA*[50] and converted to structure factors with *TRUNCATE*[51].

### Structure determination and analysis

The structure was solved by molecular substitution. The 1.7 Å crystal structure of the R292K mutant of tern N9 influenza virus NA [24] (2QWA) without the glycans or solvent molecules was used as the starting model in rigid body and coordinate refinement in *REFMAC*[52]. Refinement was continued using *PHENIX*[53] together with iterative rounds of model rebuilding using the molecular graphic package *COOT*[37]. The *PRODRG* webserver [54] was used to build the initial coordinates and stereochemical restraints of inhibitor **2**. The restraints for three β-D-mannose monomers (BMA) were taken from the *REFMAC* library [34]. Riding hydrogen atoms were added with the program *REDUCE*[55].

The structure was analyzed with *HBPLUS*[29] and *Chimera*[28]. The figures were made using *PyMOL* (Schrödinger LLC) and Liglot+ [56].

### Accession codes

The atomic coordinates and structure factors were deposited in the protein data bank under accession code 4DGR for the inhibitor complex.

## Competing interests

The authors declare that they have no competing interests.

## Authors’ contributions

WJB, BHMM and GMA designed the research, analyzed the results and wrote the manuscript. WJB, ESJ and GK designed and synthesized the inhibitor and GMA tested it. BHMM and LV crystallized the complex, solved and refined the structure, analyzed the structure and drafted the manuscript. All authors read and approved the final manuscript.
